# Hormone Signaling Regulates Nymphal Diapause in *Laodelphax striatellus* (Hemiptera: Delphacidae)

**DOI:** 10.1038/s41598-017-13879-y

**Published:** 2017-10-17

**Authors:** Yifan Zhai, Zhiming Zhang, Huanhuan Gao, Hao Chen, Meng Sun, Wenqing Zhang, Yi Yu, Li Zheng

**Affiliations:** 1 Institute of Plant Protection, Shandong Academy of Agricultural Sciences, Jinan, 250100 China; 2 Collage of Forestry, Henan Agricultural University, Zhengzhou, 450001 China; 3 State Key Laboratory of Biocontrol and School of Life Sciences, Sun Yat-sen University, Guangzhou, 510275 China

## Abstract

Diapause is a physiological adaptation that allows an organism to survive adverse environmental conditions. Diapause occurs at a specific developmental stage in each species. There are few reports regarding the molecular regulatory mechanism of nymphal diapause in *Laodelphax striatellus*, which is an important graminaceous crop pest. Our previous studies identified the conditions for nymphal diapause in this species. Here, we combined RNA sequencing transcriptomics and quantitative proteomic analyses to identify nymphal diapause-related genes and proteins. The analysis of differentially regulated genes identified four gene/protein pairs that were synchronously up-regulated, and six gene/protein pairs that were synchronously down-regulated, suggesting that these genes may regulate nymphal diapause. The up-regulated gene juvenile hormone acid methyl transferase (*JHAMT*) and the down-regulated gene cytochrome P450 monooxygenase (CYP314A1, *Shd*) were chosen for further functional studies. After knocking-down of *LsJHAMT* and *LsShd* in *vivo* by RNA interference, the titer of JH III and 20E decreased significantly, and the duration of the nymphal development period was severely altered. Thus *LsJHAMT* and *LsShd* regulated JH III and 20E titers in the hemolymph to control the nymphal diapause status. This study may lead to new information on the regulation nymphal diapause of this important agricultural insect pest.

## Introduction

Environmental conditions are not always suitable for insects. Insects have evolved multiple strategies to adapt to environmental changes, as a response to adverse environmental conditions. Diapause, an adaptive strategy occurring at a specific developmental stage in each species, enables insects to survive unfavorable seasons. Diapause is regulated through environmental and genetic factors^1^. Most insects rely on photoperiod and temperature cues to reach diapause with decreased metabolism, arrested development and increased stress resistance etc.^2^. Several recent reviews discuss regulatory features of diapause including hormonal molecular regulation^3,4^, the circadian clock^5–7^ and energy utilization^8–10^. One unifying theme for diapause in insects may be through a hormonal signalling pathway, which has been linked to diverse features of the diapause process^11^.

Many physiological processes of insects are regulated by common mechanisms that involve juvenile hormone (JH) and 20-hydroxyecdysone (20E). These two hormones play multiple physiological roles in development, reproduction, and innate immunity in insects^12^. It has also been reported that JH and 20E play critical roles in diapause regulation^13–16^. JHs are a family of insect sesquiterpenoids synthesized by the corpora allata that regulate many aspects of insect physiology. Juvenile hormone acid methyl transferase (JHAMT), the last enzyme in the biosynthetic pathway of JH, is thought to be critical for regulating JH synthesis^17^. The role of JH in the diapause was initially investigated in the larvae of *Diatraea grandiosella* ^18^, and later studies corroborated JH’s role in the regulation of diapause in other insect species^19–23^. The hormone 20E suppressed the expression of JH esterase in the fat body, but induced it in the silk glands^24^. The hormone 20E is the most active moulting hormone, and is regulated by many enzymes. A group of cytochrome P450 monooxygenases (CYPs), encoded by Halloween genes, is involved in ecdysteroidogenesis. Among these CYPs, CYP314A1 (Shd) is the enzyme responsible for mediating the conversion of ecdysone to 20E^25,26^.

The small brown planthopper (SBPH), *Laodelphax striatellus* (Hemiptera: Delphacidae), is an economically important pest insect in East Asia, *L*. *striatellus* attacks a wide range of graminaceous crops, such as rice, wheat, corn, etc.^27^. This pest not only consumes the plant’s juice, but it also transmits viral plant diseases, such as rice stripe virus and rice black-streaked dwarf virus^28^. The molecular regulatory mechanisms related to *L*. *striatellus* nymphal diapause have not been investigated. We selected diapause and non-diapause *L*. *striatellus* populations to analyze differentially expressed genes and proteins using transcriptomic and proteomic approaches. Our result suggested that *LsJHAMT* and *LsShd* play an important role in nymphal diapause. This work could thus provide the basis for more fundamental understanding on the molecular regulation of nymphal diapause in this important agricultural insect pest.

## Results

### Transcriptomic analysis of differentially expressed genes

**Figure 1:**
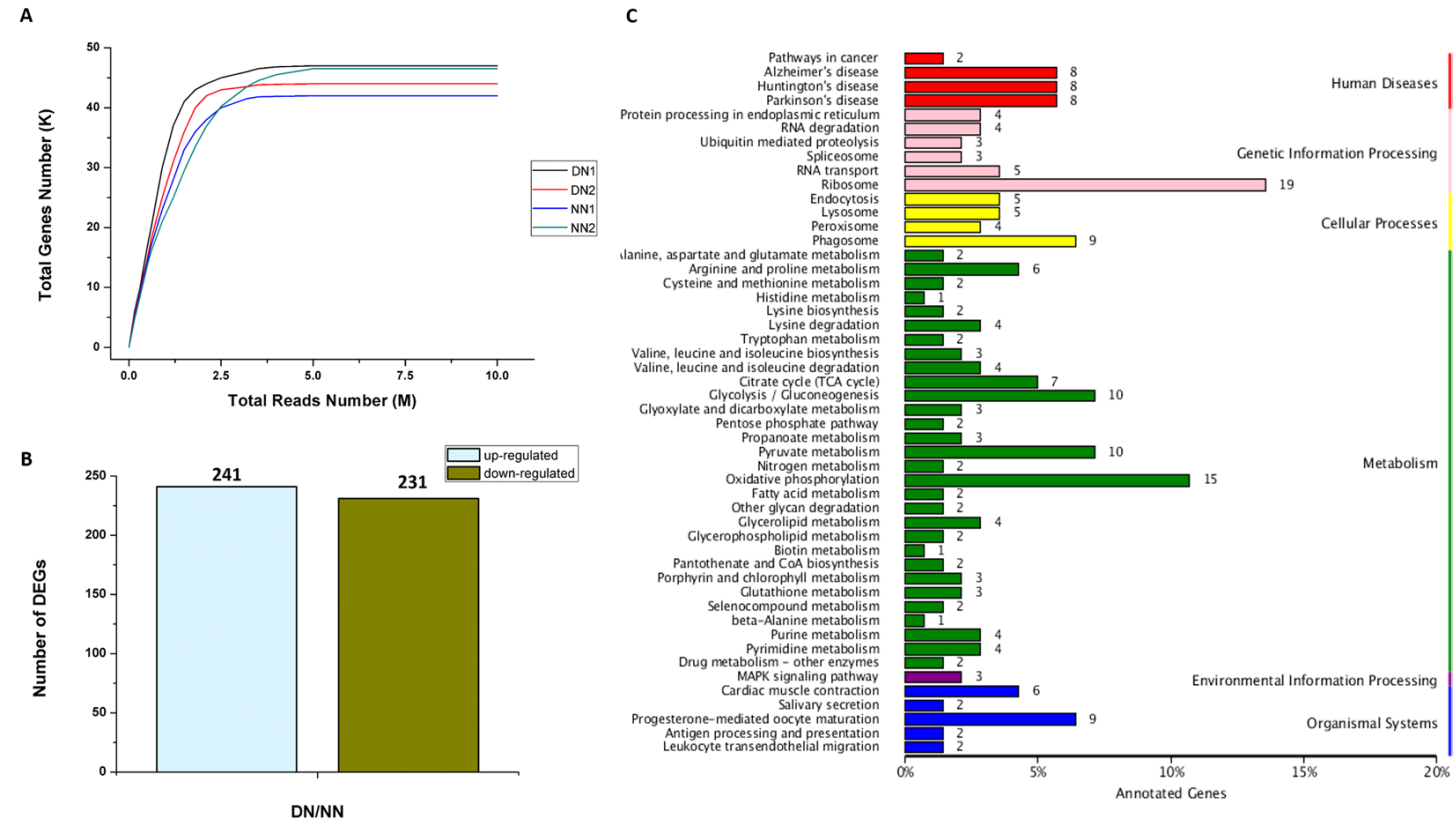
Transcriptomic analysis of the DEGs data in diapause- and non-diapause-destined nymphs. (**A**) Simulated diagram of saturation test of sequencing data. X axis indicates the number of reads (10^6^), Y axis indicates the number of detected genes (10^3^) and FPKM ≥ 0.1. (**B**) Number of significantly changed annotated DEGs, the conditions for genes was FDR ≤ 0.01 and FC ≥ 2. (**C**) The distribution of pathways of DEGs annotated in the KEGG data library.

A total of 39,796,168 clean pair-end reads were generated by Illumina sequencing, and assembled *de novo* into 63,751 unigenes, with an N_50_ length of 1,585 bp (Supplementary Table 1). Sufficient and effective genetic information was achieved in this study, as revealed by the saturation of gene number with the increase of sequenced reads (Fig. 1A). *L*. *striatellus* lacks a reference genome, so 29,217 unigenes were annotated from the databases NR (26,934), GO (10,756), COG (10,177), KOG (19,719) and the Kyoto Encyclope-dia of Genes and Genomes (KEGG, 9,816) using a cut-off e-value of 10^–5^ (Supplementary Table 2). According to the gene expression level (FPKM), the reliability of DEG was analyzed by evaluating the correlation of two biological replicates in NN and DN (Supplementary Figure 1). Based on the DEG analysis, 472 annotated genes were differentially expressed in non-diapause *vs*. diapause nymphs, 241 and 231 genes were up- and down-regulated, respectively (Fig. 1B). To identify pathways regulated during diapause, we performed a pathway clustering analysis using the KEGG database. The results showed that most of the DEGs correlated to metabolic processes, including carbohydrate and amino acid metabolism (Fig. 1C, Supplementary Figure 2).

### Global changes at the protein levels

**Figure 2:**
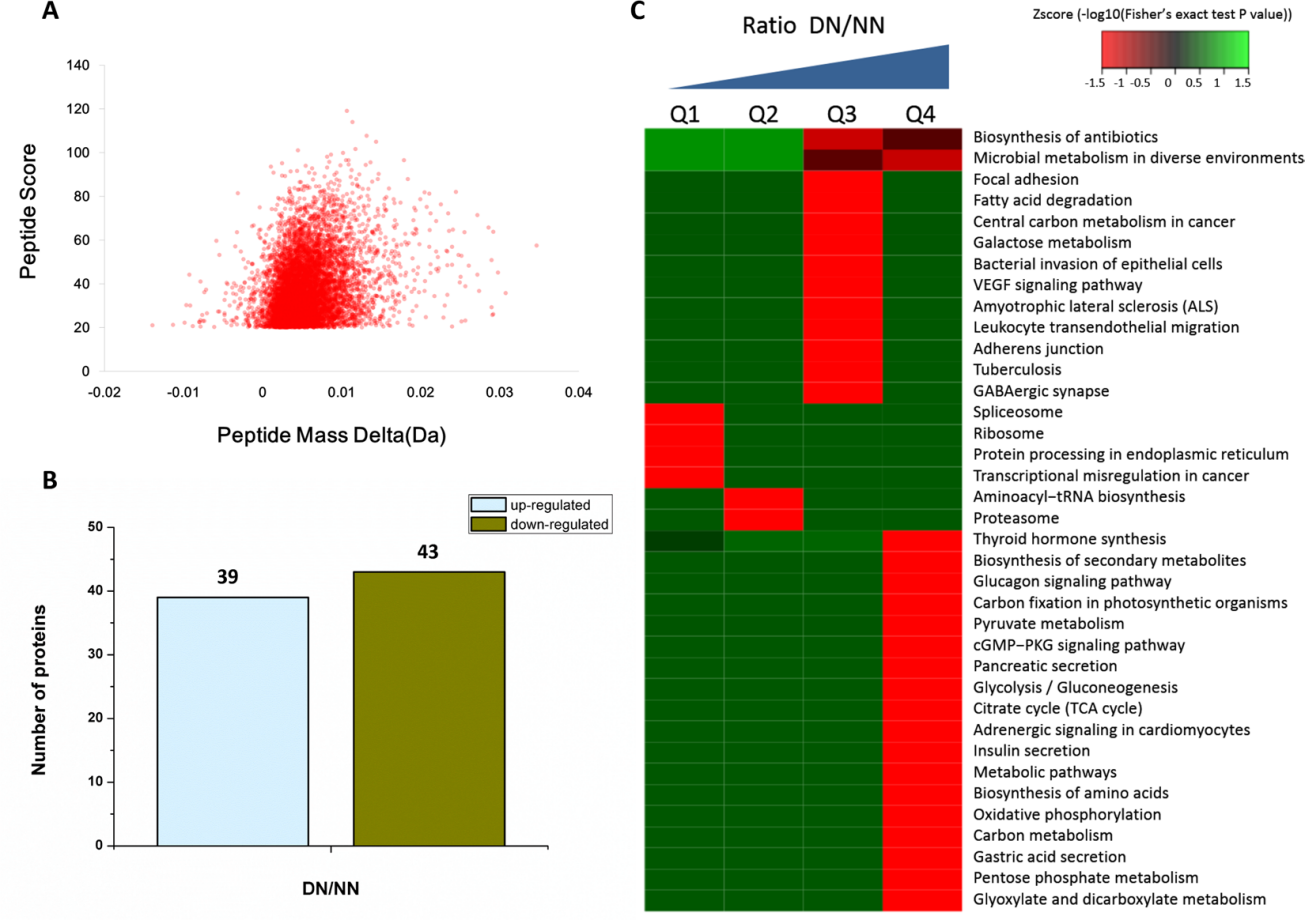
TMT analysis of the differentially expressed proteins data in diapause- and non-diapause destined nymphs. (**A**) Quality control validation of MS data, mass error distribution of all identified peptides. (**B**) Number of differentially quantified proteins. On the basis of duplicate biological replications analyses, only proteins that changed ≥1.3-fold in relative ratios (*p* < 0.05) were considered. (**C**) Enrichment and clustering analysis of the quantitative proteomics data sets based on KEGG pathway database.

Proteins from the NN and DN were used for tandem mass tags (TMT) labeling and HPLC fractionation followed by high-resolution LC-MS/MS analysis, quantitative global proteome analysis was then performed. First the MS data were validated, the distribution of mass error was near zero and most of them were less than 0.02 Da, which meant that the mass accuracy of the MS data fit the requirement (Fig. 2A). The length of most peptides was between 8 and 16 amino acids, which is in agreement with the property of tryptic peptides (Supplementary Figure 3), and with the relative quantitative correlation of the two biological replicates of the proteome (Supplementary Figure 4). In the samples, 1,421 proteins were annotated from *Uniprot_Neohemiptera* insects databases, from which 1,329 proteins could be quantified (Table 1). When we set a quantification ratio >1.3 as the threshold for up –regulation, and a quantification ratio <0.77 as the threshold for down regulation, we obtained 82 annotated proteins that were statistically significant responders (*p* < 0.05). A total of 39 and 43 proteins were up- and down-regulated, respectively (Fig. 2B, Supplementary Table 3). According to the KEGG classification, we performed clustering analyses by dividing all significantly changed proteins into four quantiles (Q1–Q4) according to DN/NN ratios (Q1: <0.77; Q2: 0.77–1; Q3: 1–1.3; Q4: >1.3) to see the biological functions of the proteins with large changing ratios (>1.3 or <0.77) or with relatively small changing ratios (0.77–1 or 1–1.3) upon diapause treatment. The KEGG pathway analysis of the quantitatively changed proteins during diapause identified a number of vital pathways. Some carbohydrate metabolism, including glyoxylate and dicarboxylate metabolism, pentose phosphate metabolism, the citrate cycle, and glycolysis/gluconeogenesis decreased during diapause (Fig. 2C).

**Table 1:** The High Confidence Quantitative Proteome: Size and Features.

Identified nonredundant peptides	69,188
Identified nonredundant proteins	1,421
Quantifiable Proteins (with unique peptides >0 + non unique >1)	1,329
Differential expressed proteins (*p*-value < 0.05, ratio ≥1.3 or ≤0.77)	82
Up-regulated Proteins	39
Down-regulated Proteins	43

### Validation of differentially expressed genes

To validate the profiles of genes expression, we selected 30 DEGs (16 up-regulated and 14 down-regulated genes) for qRT-PCR, using two reference genes (A: *ARF*; B: *EF-1*). The results showed that 27 of the 30 DEGs (except in C41099, C10990 and C34143; Supplementary Figure 5A) were up- or down-regulated according to the results of the transcriptomic analysis. Only four genes (C41099, C39772, C10990 and C35294) in Supplementary Figure 5B were not confirmed from the results of the DEG analysis. The gene expression profiles were similar regardless of the reference gene used. We thus conclude that 83.33% of genes analyzed by qRT-PCR were in accordance with the electronic data of transcriptomic analysis.

### The correlation between mRNA and protein expression profiles

**Figure 3:**
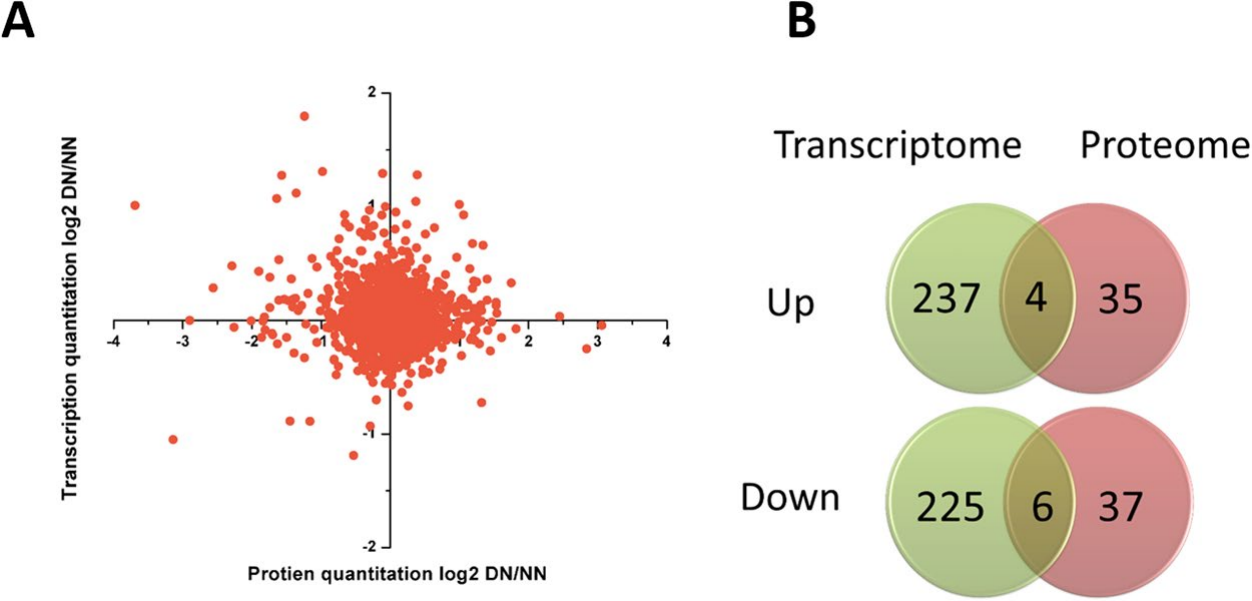
Combined analyses of the TMT and DEGs data. (**A**) The relationship of transcriptome and proteome. (**B**) Venn diagrams of differentially expressed genes/proteins from TMT and DEGs analyses.

Using the quantitative proteomic and transcriptomic data sets, we analyzed the relative quantitative correlation of transcriptomes and proteomes (Fig. 3A). Genes whose mRNA and protein levels were differentially expressed, at *p* < 0.05 for protein and false discovery rate (FDR) < 0.01 for mRNA, were selected for analysis. At the protein and mRNA levels, we identified four genes with overlapping gene and protein up regulation group and six genes with overlapping gene and protein down regulation (Fig. 3B, Supplementary Table 4). From these genes, *JHAMT* and cytochrome P450 monooxygenase (*CYP314A1*) were selected for further functional studies (Supplementary Figure 6).

### Effect of knocking-down *LsJHAMT* and *LsShd* on nymph performance

**Figure 4:**
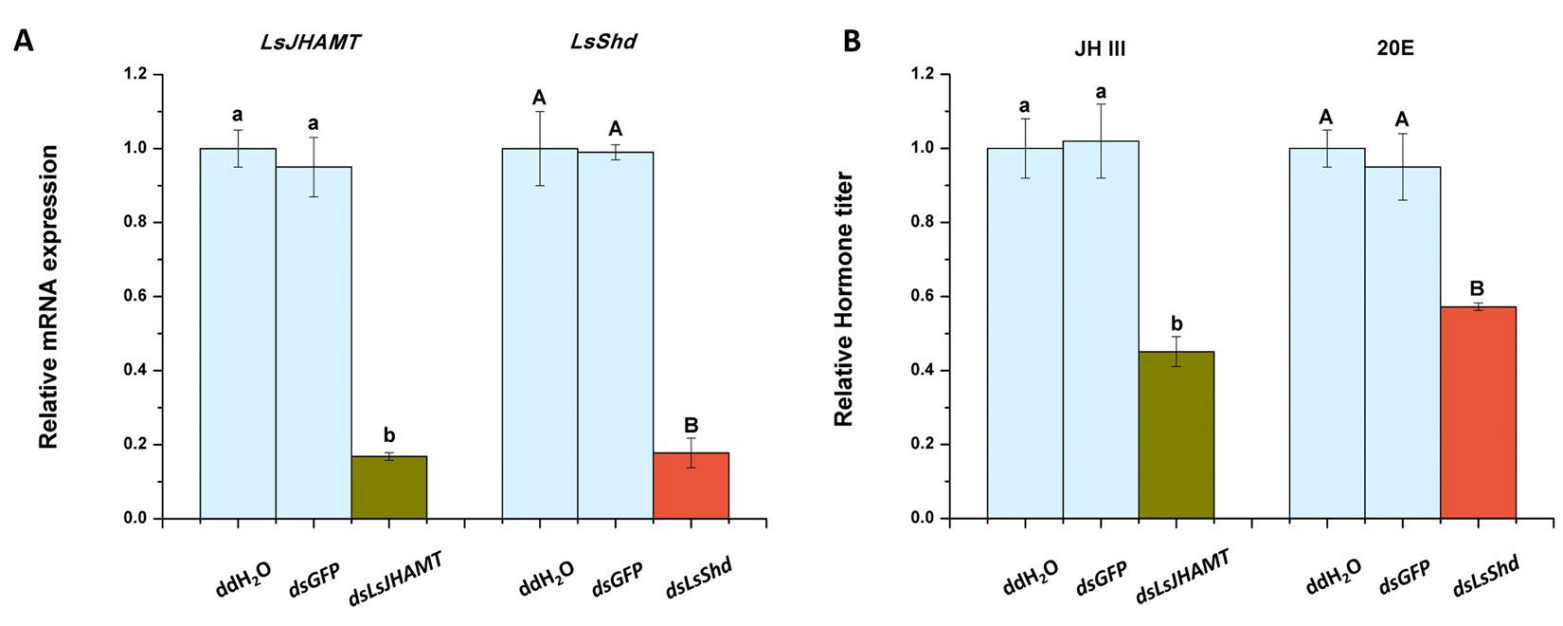
Effect of *LsJHAMT* or *LsShd* knockdown on gene expression and hormone titer. (**A**) The transcript levels of *LsJHAMT* or *LsShd* after injection by qRT-PCR. (**B**) The JH III and 20E titer was analyzed at 48 h post-injection. The data represent the mean values ± SEM (n = 3), and the values in the columns followed by different letters denote a significant difference (*p* < 0.05, Tukey’s post hoc test).

We used a microinjection method for RNA interference, to evaluate the effect of knocking-down *LsJHAMT* and *LsShd* during diapause or during normal development. At 48 h after injection of *dsLsJHAMT* or *dsLsShd*, the knockdown efficiency of *LsJHAMT* and *LsShd* was 83.19% and 82.24%, respectively. (Fig. 4A). JH III and 20E titer analyses showed that the hormone levels were reduced (Fig. 4B, Supplementary Figure 7). These results indicated that RNA interference was effective.

**Figure 5:**
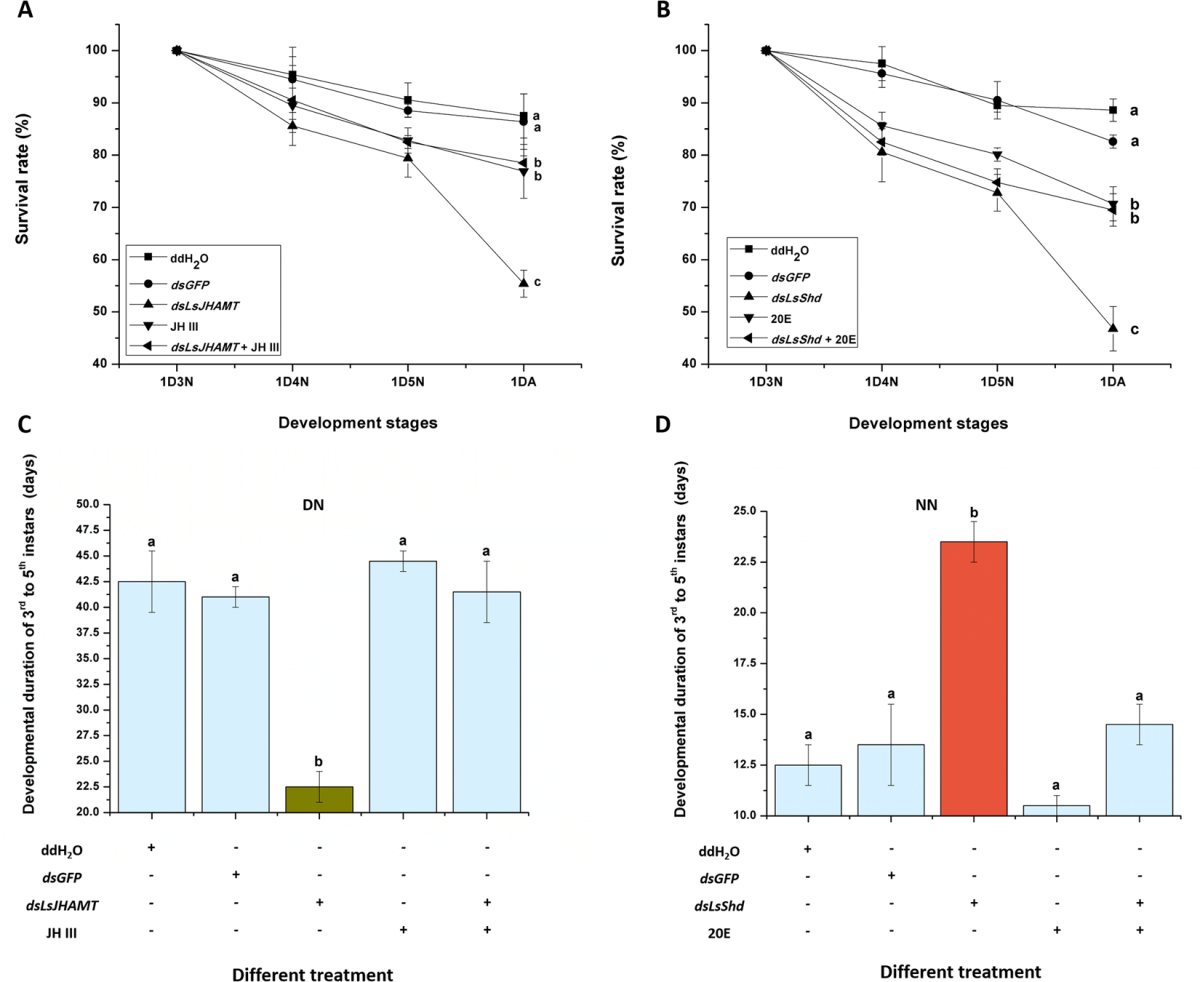
Effect of different treatment on nymphal survival rates and developmental duration. (**A**) The survival rates of diapause nymphs from 3^rd^ instar to the initiation of adult stage. (**B**) The survival rates of non-diapause nymphs. 1D3N, 1D4N, 1D5N and 1DA represent first day of 3^rd^, 4^th^, 5^th^ instar nymphs and emerged adults. (**C**) The developmental duration of diapause nymphs. (**D**) The developmental duration of non-diapause nymphs. The data represent the mean values ± SEM (n = 3), and the values in the columns followed by different letters denote a significant difference (*p* < 0.05, Tukey’s post hoc test).

Injection with *dsLsJHAMT* or *dsLsShd* significantly decreased nymphal survival rate, the nymphal period from 3^rd^ to 5^th^ instar after injection, the nymphal survival rate separately decreased to 55.4% and 46.8%. By contrast, over 85% and 80% of the nymphs injected with water or *dsGFP* survived (Fig. 5A,B). The duration of diapause in nymphs injected with *dsLsJHAMT* was notably shorter than other treatments at 20 °C under short day-length (10 L: 14D). When JH III was injected together with *dsLsJHAMT*, the nymphs period from 3^rd^ instar to the initiation of adult eclosion was significantly increased (Fig. 5C). However, the opposite trend was observed when *LsShd* was knocked-down in non-diapause nymphs at 20 °C under long day-length (16 L: 8D). The average duration of nymphs injected with *dsLsShd* to adult eclosion was 23.5 days, which was significantly longer than the mean periods of other treatments. When 20E was injected together with *dsLsShd*, the period from 3^rd^ instar to the initiation of adult eclosion was significantly shortened (Fig. 5D).

## Discussion

Integration of “multi-omics” approaches has already been applied in many fields, in order to improve the credibility of data sets, obtain multilayered pictures of regulatory processes and good for carrying out the follow-up^29–31^. RNA-sequencing (RNA-Seq) transcriptomic and quantitative proteomics are two powerful approaches for large-scale research of translationally and post-translationally regulated networks. Stable isotope labeling has been applied to increase the accuracy of quantitative results. Proteomics using mass spectrometry with TMT is a reliable technology for quantitative proteome analysis^32,33^.

In our study, KEGG pathway classification analysis of combined TMT proteomics and RNA-Seq transcriptomic data, showed that carbohydrate metabolism, including glyoxylate and dicarboxylate metabolism, pentose phosphate metabolism, the citrate cycle, and glycolysis/gluconeogenesis decreased during diapause (Figs 1C and 2C). Sugars, the tricarboxylic acid cycle, and other carbohydrate metabolites may be closely related to the diapause process, and it is also possible that the TCA cycle may be a checkpoint for regulating different forms of animal dormancy. We found 4 gene/protein pairs that were synchronously up-regulated: arginine kinase (R4WCV2), annexin (A0A0A9XQG0), *JHAMT* (A0A0K1IJN8) and tropomyosin (V5JDH8). In addition, we found 6 gene/protein pairs that were synchronously down-regulated: calreticulin (R4WHW2), 60S ribosomal protein (G8CV16), ATP synthase (A0A0E3DQZ8), *Shd* (M9SV74), elongation factor 2 (A0A0A9W869) and Hsp 70 (V5TGF4) (Supplementary Table 4). Similar to the previous report^34^, the relative of change in mRNA and protein levels was different. It is well known that unlike quiescence, diapause is a hormonally-regulated physiological process, so *LsJHAMT* and *LsShd* were selected for further functional studies.

JH and 20E are commonly known for coordinating insect development and growth. Diapause regulation by the two hormones has been reported in different kinds of insects, including Lepidoptera, Diptera, Hymenoptera^13,15,35^. Previous studies demonstrated that a high JH titer inhibits ecdysone secretion during diapause maintenance, and that the JH titer decreases significantly during late diapause^36–38^. Several reports on *Drosophila melanogaster*, *Tribolium castaneum*, *Apis mellifera*, and *Bombyx mori* indicate that *JHAMT* catalyzes the final step of JH biosynthesis^17,39–41^. The Halloween gene Shd is the enzyme responsible for mediating the conversion of ecdysone (E) to 20E^26,42^. *JHAMT* and *Shd* plays an important role in JH III biosynthesis and in the ecdysteroid pathways. A positive correlation between *JHAMT* expression and JH titer in *D*. *melanogaster* has been found, and the same correlations have been found between *Shd* expression levels and 20E in *L*. *striatellus* ^34,43^. Our results also showed that the concentration of JH III and 20E decreased after *dsLsJHAMT* and *LsShd* injection, respectively (Fig. 4B). In order to directly test for hormones changes during diapause or normal development, we analyzed the hormones titers in diapause and non-diapause nymphs. The JH III titer was notably higher in diapause nymphs, however the opposite result of 20E was observed (Supplementary Figure 8).

Diapause is a complex biological process. Upstream signaling factors regulate diapause-related genes expression, for example, photoperiodic signals are received by the light-sensitive cells within the brain^44^. There are many morphological indications of diapause in insects, such as eye-spots in the postgenal region of the *H*. *armigera* pupa during movement^45^. However, there are no evident morphological features that reveal diapause and non-diapause of *L*. *striatellus* nymphs. In the species diapause is determined by the duration of development. Diapause evaluation is based on the proportion of nymphs remaining as 3^rd^ and 4^th^ instar for seven days, after comparable control nymphs have completed emergence^46^. Our previous studies also showed that the developmental duration of the 3^rd^ and 4^th^ instars was longer during the short day-length (10 L: 14D) at 20 °C than other photoperiods. In the present work, we found that the average duration from *dsLsJHAMT* injection to adult eclosion was 22.5 days at 20 °C under short day-length (10 L: 14D), which significantly shorter than the mean periods of other treatments, and that the survival rate was 55.4% significantly lower than others. When JH III was injected together with *dsLsJHAMT*, insects remained as nymphs significantly longed (Fig. 5A and C). However, the opposite trend was observed when *LsShd* was knock-down in the non-diapause nymphs at 20 °C under long day-length (16 L: 8D). The duration of diapause nymphal development was notably longer. When 20E was added, the duration of nymph stages from 3^rd^ instar to the initiation of adult eclosion were significantly shortened (Fig. 5D). These results suggest that *LsJHAMT* and *LsShd* may regulate nymphal development by controlling the accumulation of hormone in hemolymph.

**Figure 6:**
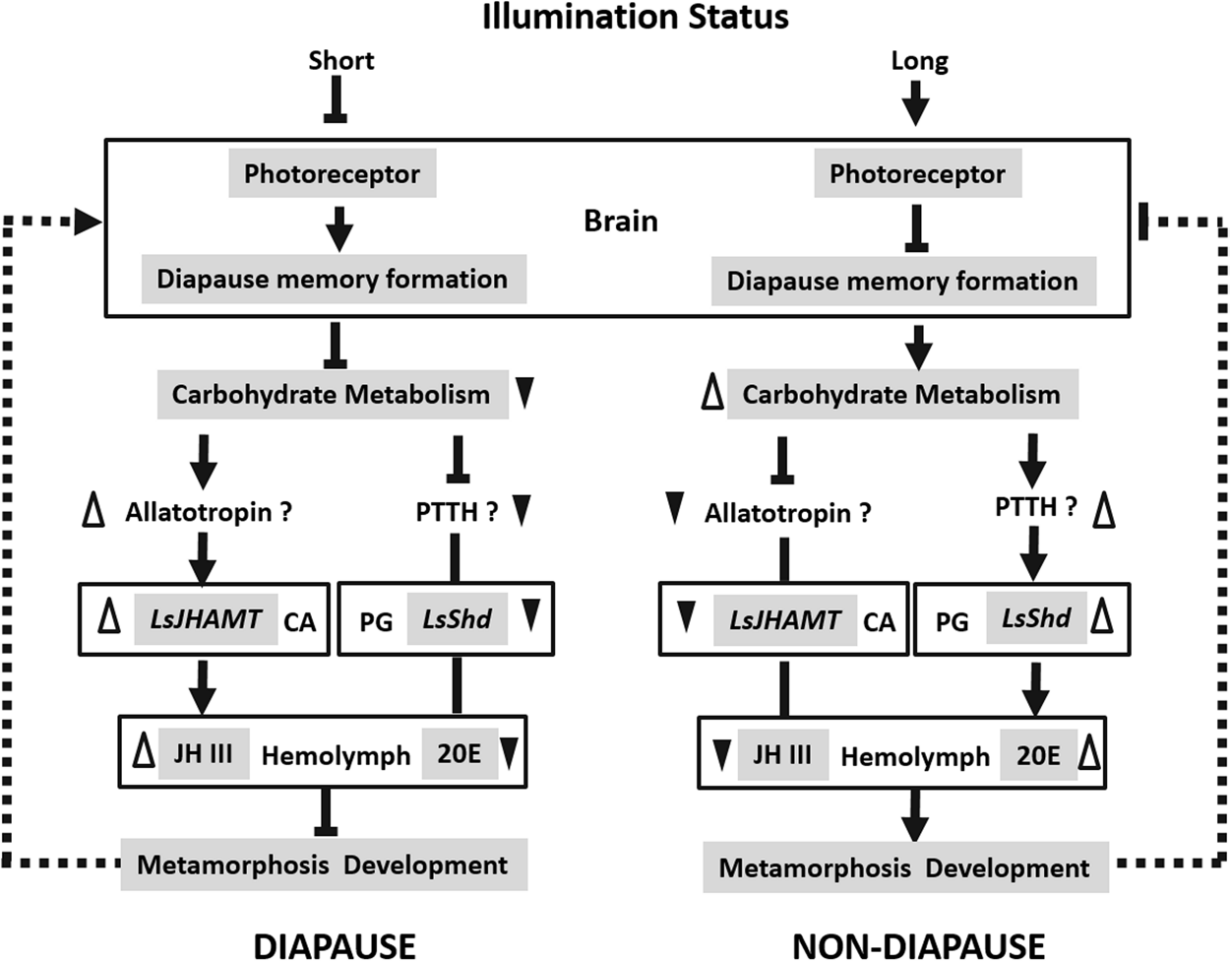
Proposed model of the different photoperiodic signals regulate the nymphal diapause. Short illumination status decreased photoreceptor activity, and the photoperiodic information will subsequently be stored by a special memory formation process in the brain. The carbohydrate metabolism was repressed, which stimulates the JH III synthesis in CA (corpora allata). Instead, the 20E were down-regulated in PG (prothoracic gland). In this state, weak metamorphosis development (diapause) may stimulates photoreceptor activity by a feedback loop mechanism. Long illumination status results in opposite effects. The model provides an explanation for different photoperiodic signals interacts with hormones (JH III or 20E) titers to regulate the nymphal diapause. PTTH: prothoracicotropic hormone.

In conclusion we used a combination of multi-omics data analysis in order to identify the putative effectors regulating nymph diapause in *L*. *striatellus*. The integrated quantitation and comparison of mRNA and protein abundances revealed extensive translational and post-translational regulation of diapause. Moreover, we confirmed that *LsJHAMT* and *LsShd* could regulate JH III and 20E titers, respectively, in the hemolymph so as to control the nymphal diapause status. We proposed a possible model to explain how different photoperiod signals interacts with JH III and 20E to regulate nymphal diapause in *L*. *striatellus* (Fig. 6). There are still many issues in the model to be studied in the future, such as which factors regulate *LsJHAMT* and *LsShd* expression. However, the present results will offer new insights into the studies on the nymphal diapause and contribute to a comprehensive view of insect diapause.

## Methods

### Insect Rearing

We used a *L*. *striatellus* colony that was originally obtained from Shandong Rice Research Institute (SRRI; Shandong, China) in 2010. This strain was reared in a continuous laboratory culture on fresh rice seedlings, and maintained in the laboratory at 25 ± 1 °C with 70–80% humidity and under a 16 L: 8D daylight cycle.

Newly hatched 1^st^ instar nymphs were reared on fresh rice seedlings at 20 °C under long day-length (16 L: 8D), which resulted in all individuals continuing through direct development (non-diapause), or 10 L:14D, resulting in substantially all individuals entering nymphal diapause, and developmental delay often characterized nymph population diapause^47^. Two biological replicate samples were stored at −80 °C until use, and each sample was divided into two parts for global proteomics and whole-genome transcriptomics analyses.

### RNA-Seq transcriptomic analysis

Total RNA was extracted using the E.Z.N.A.^®^ Total RNA Kit II (Omega, USA) according to the manufacturer’s protocol. The samples were treated with DNase, and the quantity and quality of each RNA sample was assessed using micro volume spectrophotometer (NanoDrop 2000, Thermo) and a Bioanalyzer (Aglient 2100, Life Tech), respectively. Only the RNA samples with 260: 230 ratios from 2.0 to 2.5, with 260: 280 ratios from 1.9 to 2.1, and with an RNA integrity number >8.0, were used for subsequent analysis.

The construction of cDNA libraries and RNA-Seq were performed by the Biomarker Biotechnology Corporation (Beijing, China). According to the Illumina manufacturer’ s instructions, poly(A)^+^ RNA was purified from 10 μg of pooled total RNA using oligo (dT) magnetic beads and fragmented into short sequences in the presence of fragmentation buffer. The cleaved mRNA was transcribed with random hexamers, and then second-strand cDNA synthesis was performed. After the purification of cDNA using AMPure XP (Beckman Coulter, USA) beads, end repair and ligation of adaptors, the products were amplified by PCR to create a cDNA library. Each cDNA library was sequenced using the Illumina sequencing platform (Hiseq. 2500). The raw reads from the images were generated using a Solexa GA pipeline 1.6 sequencing by synthesis. After removal of low-quality reads containing primer or adaptor sequences, and trimming of read lengths using SeqClean, high-quality reads were considered as clean data, with an identity value of 95% and a coverage length of 125 bp, and were assembled de novo using Trinity software and clustered using the De Bruijn graph algorithm. The unigene sequences were generated after unbinding the De Bruijn graph. According to the alignment with sequences in the unigene library, the mapped reads in the clean data of each sample were used for quality evaluation of the transcriptome sequencing library. Quality evaluation included randomness of mRNA fragmentation and saturation tests of sequencing data. Reads sequenced from each sample were aligned with the unigene library using Bowtie^48^. To obtain relative expression levels in each sample, fragments per kilobase of transcript per million mapped reads (FPKM) in each sample were counted and combined with RSEM^49^. To ensure the reliability of differential expression of genes, the Pearson’s Correlation Coefficient (r) was deeded as evaluation indicator of correlation of two biological replicates^50^. DEGs were identified by the DESeq package using the Benjamini-Hochberg method. The global FDR < 0.01 and fold change of the FPKM value of two compared goups ≥2 were used as the thresholds to determine significant differences in gene expression.

### TMT quantitative proteomic analysis

Samples from non-diapause and diapause nymphs (4^th^ instars) were ground into powder in liquid nitrogen, homogenized in lysis buffer (8 M urea, 1% Triton-100, 65 mM DTT and 0.1% Protease Inhibitor Cocktail III), and then centrifuged at 12,000 rpm at 4 °C. The supernatant was precipitated with cold 15% trichloroacetic acid/acetone for 2 h at −20 °C. After centrifugation at 12,000 rpm at 4 °C for 10 min, the remaining precipitate was washed with cold acetone three times, then the protein was redissolved in buffer (8 M urea, 100 mM TEAB, pH 8.0) and the protein concentration was determined using a 2-D Quant kit (GE Healthcare, USA).

Total protein (100 μg) solution was reduced with 10 mM DTT for 1 h at 37 °C and alkylated with 20 mM IAA for 45 min at room temperature in the dark. Proteins were then diluted by adding 100 mM TEAB to reach a urea concentration lower than 2 M. Finally, trypsin was added at 1:50 trypsin-to-protein mass ratio for the first digestion overnight, and at 1:100 trypsin-to-protein mass ratio for a second 4 h-digestion. After trypsin digestion, peptides were desalted using a Strata X C18 SPE column (Phenomenex) and dried under vacuum. Peptides were reconstituted in 0.5 M TEAB and labeled with 6-plex TMT reagents (ThermoFisher Scientific, USA) as follows: diapause nymph-1 (DN1), 126; diapause nymph-2 (DN2), 127; non-diapause nymph-1 (NN1), 128; and non-diapause nymph-2 (NN2), 129. The four tagged peptide samples were then incubated for 2 h at room temperature, pooled, desalted, and dried by vacuum centrifugation.

The samples were then fractionated by high pH reverse-phase HPLC using an Agilent 300Extend C18 column (5 μm particles, 4.6 mm ID, 250 mm length). Briefly, peptides were first separated with a gradient of 2% to 60% acetonitrile in 10 mM ammonium bicarbonate (pH 10) for over 80 min into 80 fractions. The peptides were then combined into 18 fractions and dried by vacuum centrifugation. Two independent biological experiments each with three technical replicates were performed. Then all the MS/MS data were processed using Mascot search engine (v.2.3.0) with the target-decoy database searching strategy^51^ against *Uniprot_Neohemiptera*.*fasta* database. Trypsin/P was specified as cleavage enzyme allowing up to two missing cleavages. Mass error was set to 10 ppm for precursor ions and 0.02 Da for fragment ions. Carbamidomethyl on Cys, TMT-6plex (N-term) and TMT-6plex (K) were specified as fixed modification and oxidation on Met was specified as variable modifications. The FDR was adjusted to <0.01 and peptide ion score was set to >20.

### Quantitative real-time PCR analysis

The primers used for real-time PCR are listed in Supplementary Table 5. The synthesized first-strand cDNA was amplified by PCR in 10 μL reaction mixtures using a Light Cycler 480 system (Roche, USA), and *ADP-ribosylation factor* (*ARF*) and *elongation factor-1* (*EF-1*) genes were used as the internal control genes^52^. After amplifications, melting curve analysis was performed in triplicate, and the results were averaged. The quantitative variation was calculated from three independent biological samples using the relative quantitative 2^−ΔΔCT^ method.

### RNA interference and bioassays

The dsRNA of a target gene was produced using specific primers (Supplementary Table 5) conjugated with the T7 RiboMAX™ Express RNAi System (Promega, USA) promoter. After synthesis, the *dsLsJHAMT* (439 bp), *dsLsShd* (470 bp), and *dsGFP* (414 bp) were quantified by using a micro-volume spectrophotometer (NanoDrop 2000, ThermoFisher) and maintained at −80 °C until use. The sequence was verified by sequencing (Sangon Biotech, Shanghai, China).

Before injection, the dsRNA and phenol red solution were mixed for observation^53,54^. First day third-instar nymphs were immobilized on the agarose injection plate with the ventral side upward, under CO_2_ anesthesia. Forty nL of the purified dsRNA (200 ng), JH III (50 ng), 20E (50 ng) or ddH_2_O were slowly injected on one side of the metathorax using the Nanoject II (Drummond, USA). The injected individuals were placed in a glass tube (length: 200 mm; diameter: 25 mm) on fresh rice seedlings for further observation. Data on developmental duration were recorded every day until the adult emerged.

### Quantitative determination of hormone

JH III and 20E analyses were modified from methods described previously^55^. In brief, *L*. *striatellus* samples were separately ground in grinder and ultra sonicated with methanol and isooctane. After centrifugation at 12,000 g for 10 min, the upper layer was transferred into a test tube, the ultrasound-assisted extraction was repeated twice. The combined extracts were evaporated to dryness in a 40 °C water-bath under a stream of nitrogen. The residue was reconstituted in methanol, then transferred to injection vials and analyzed using HPLC-MS/MS, (Agilent 6420; Waldbronn, Germany). JH III and 20E were separated using gradient elution and the two hormones titers were expressed as ng per mg body weight.

### Statistical analysis

Results were expressed as means ± SE, and were analyzed by one-way analysis of variance (α = 0.05) test, followed by the Tukey–Kramer test, using SPSS for Windows (SPSS, Chicago, IL, USA).

## Electronic supplementary material

Supplementary information
