# Supplementary material for: Hormone Signaling Regulates Nymphal Diapause in Laodelphax striatellus (Hemiptera: Delphacidae)
Source: Sci Rep. 2017 Oct 17;7:13370. doi: 10.1038/s41598-017-13879-y (PMC5645394; doi:10.1038/s41598-017-13879-y)
Supplement: Supplementary file 1 — Supplementary information [file 41598_2017_13879_MOESM1_ESM.pdf]

Hormone Signaling Regulates Nymphal Diapause in *Laodelphax striatellus* (Hemiptera: Delphacidae)

Yifan Zhai[1*], Zhiming Zhang[2], Huanhuan Gao[1], Hao Chen[1], Meng Sun[1], Wenqing Zhang[3], Yi Yu[1], Li Zheng[1*]

[1] Institute of Plant Protection, Shandong Academy of Agricultural Sciences, Jinan 250100, China

[2] Collage of Forestry, Henan Agricultural University, Zhengzhou 450001, China

[3] State Key Laboratory of Biocontrol and School of Life Sciences, Sun Yat-sen University, Guangzhou 510275, China.

* Corresponding author:

E-mail: zhengli64@126.com, zyifan@saas.ac.cn

Tel: +86 0531 83179902,

Fax: +86 0531 83179218.

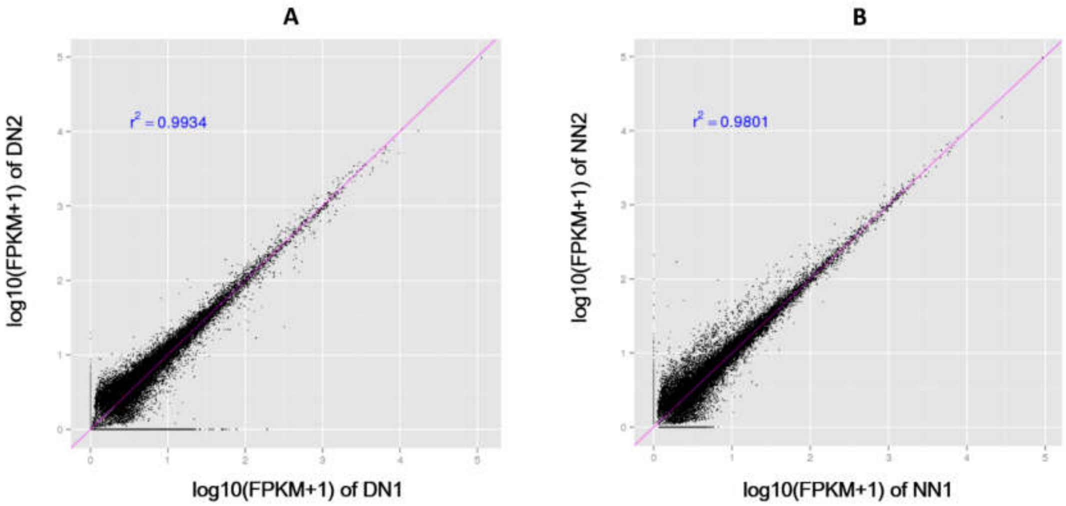

Supplementary Figure 1. Correlation plot of two biological replicates. (A) Diapause nymphs; (B) Non-diapause nymphs. Pearson's correlation coefficient (r^2) as evaluation indexes of biological repeatability and correlation.

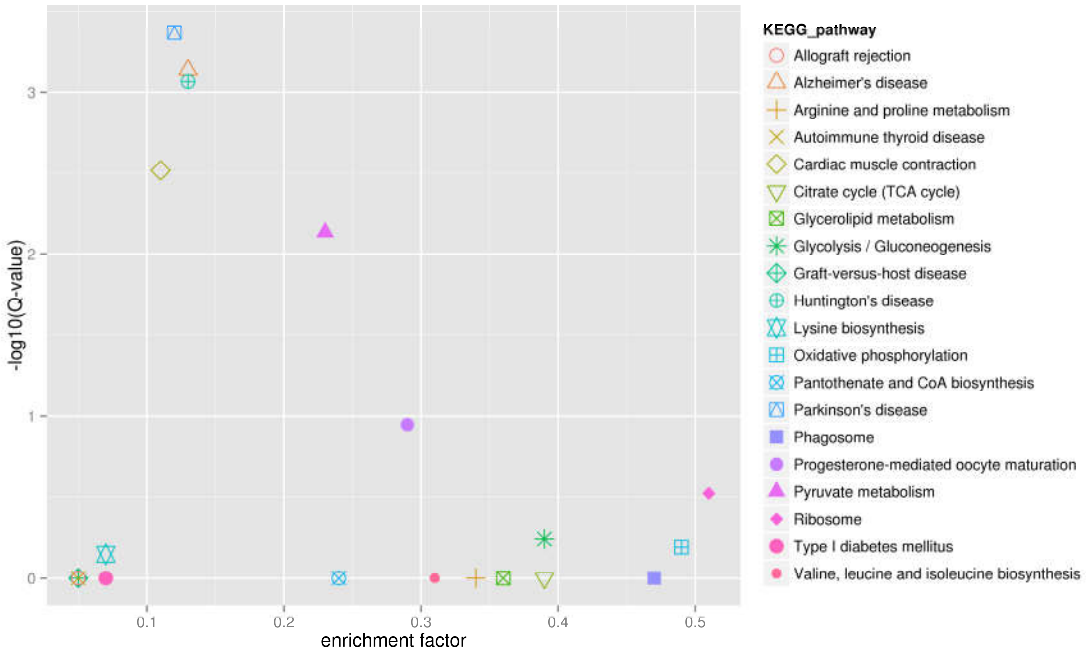

Supplementary Figure 2. The scatter diagram of DEGs pathway enrichment analysis.

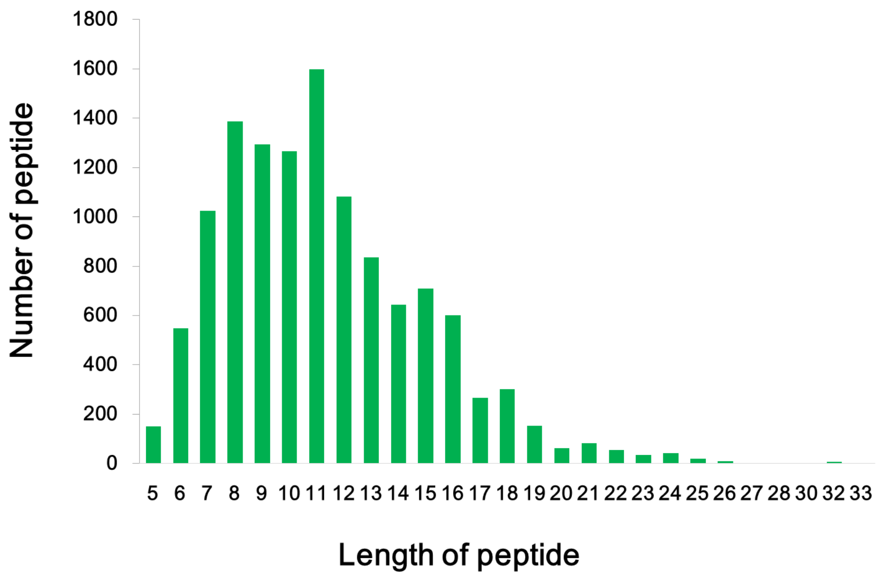

Supplementary Figure 3. The length of most peptides distributed between 8 and 16, which agree with the property of tryptic peptides

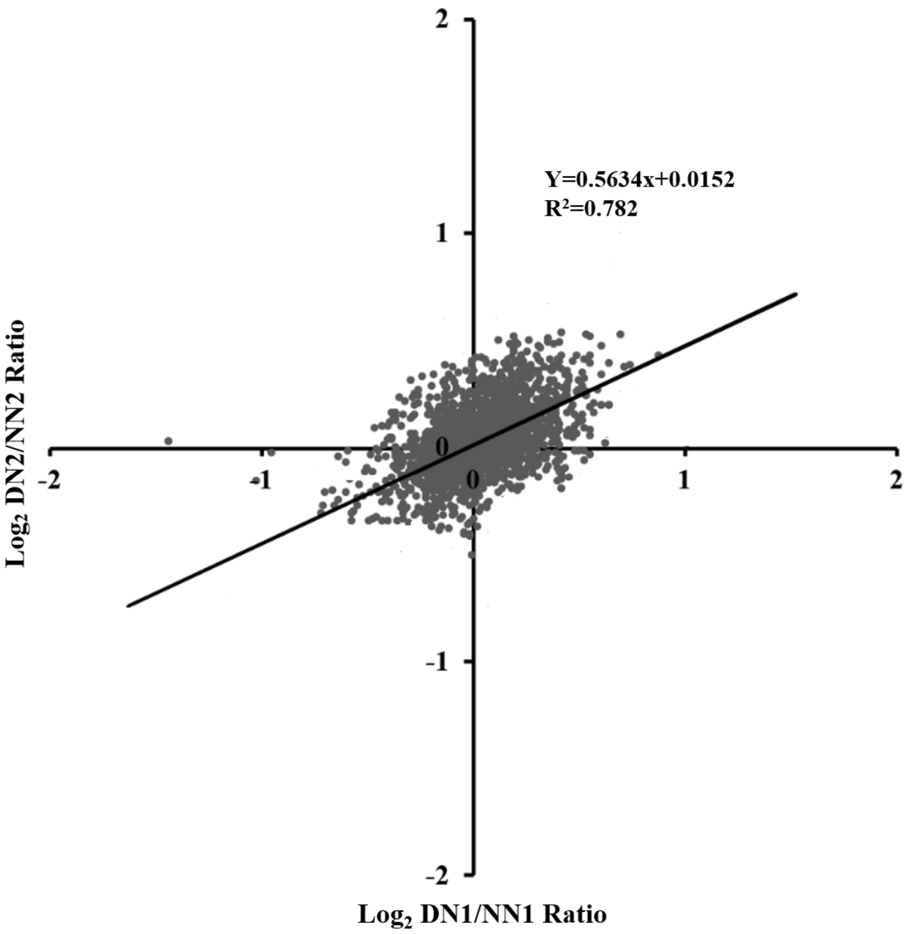

Supplementary Figure 4. The relative quantitative correlation of the two biological replicates of the proteome.

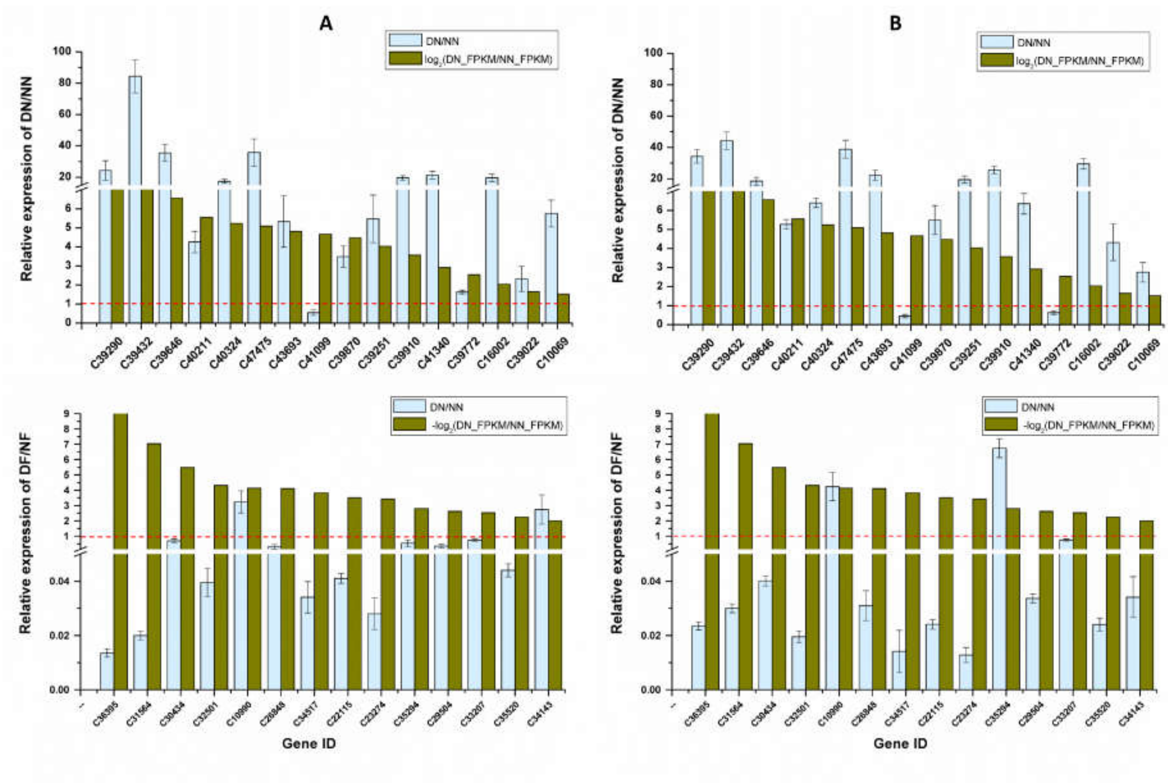

Supplementary Figure 5. Validation of differentially expressed genes by qRT-PCR. Thirty DEGs were selected. (A) *ARF* was a reference gene. (B) *EF-1* was a reference gene. The cyan bars represent the qRT-PCR results, presented as the mean ± SE (n = 3), and the dark yellow bars represent the transcriptomic analysis results. A ratio of the qRT-PCR results less/more than 1 indicated that the gene expression pattern shown by qRT-PCR was inconsistent with that obtained from the transcriptomic analysis, respectively.

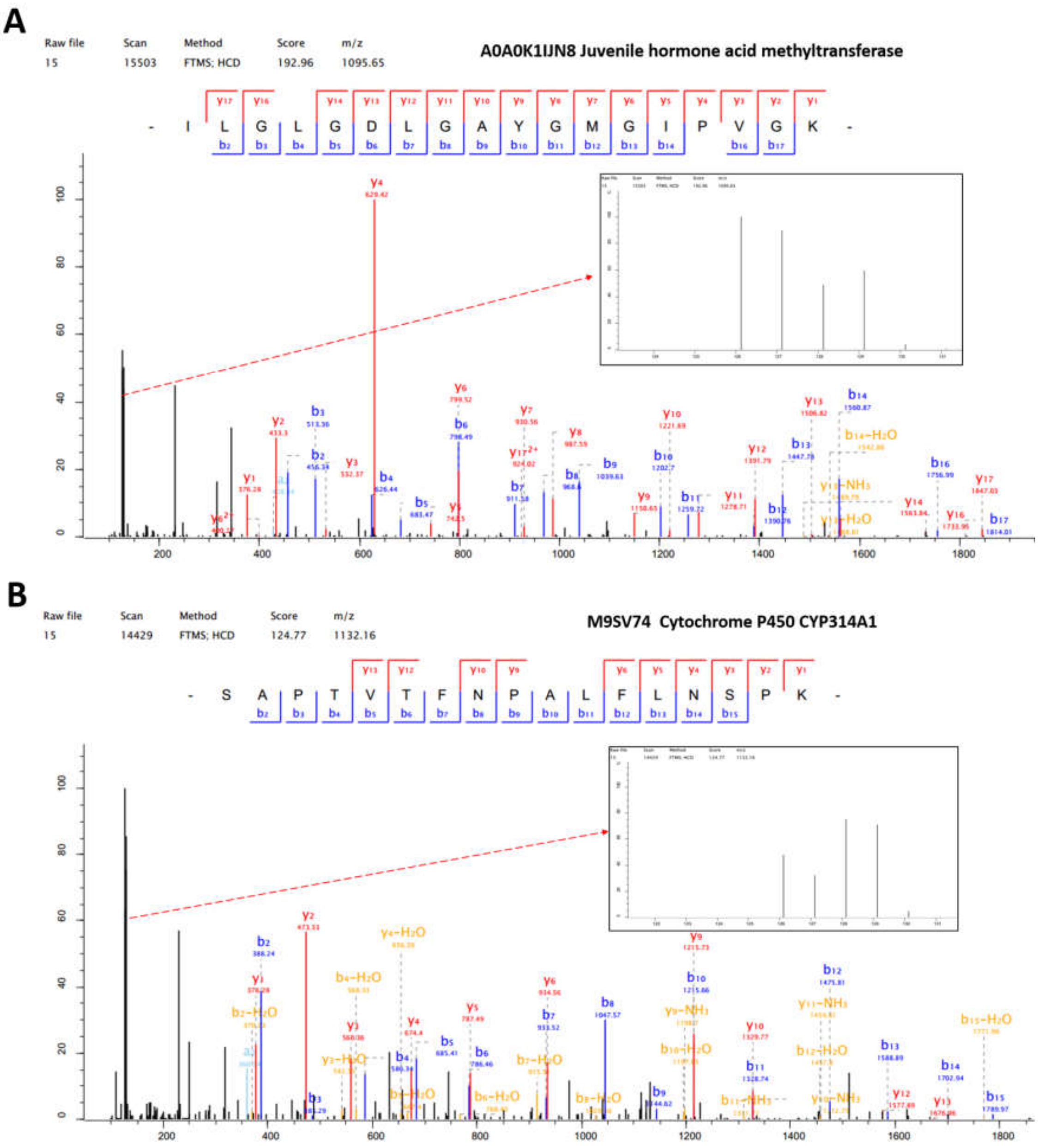

Supplementary Figure 6. The representative MS/MS spectra of up-regulated protein JHAMT and down-regulated protein CYP314A1.

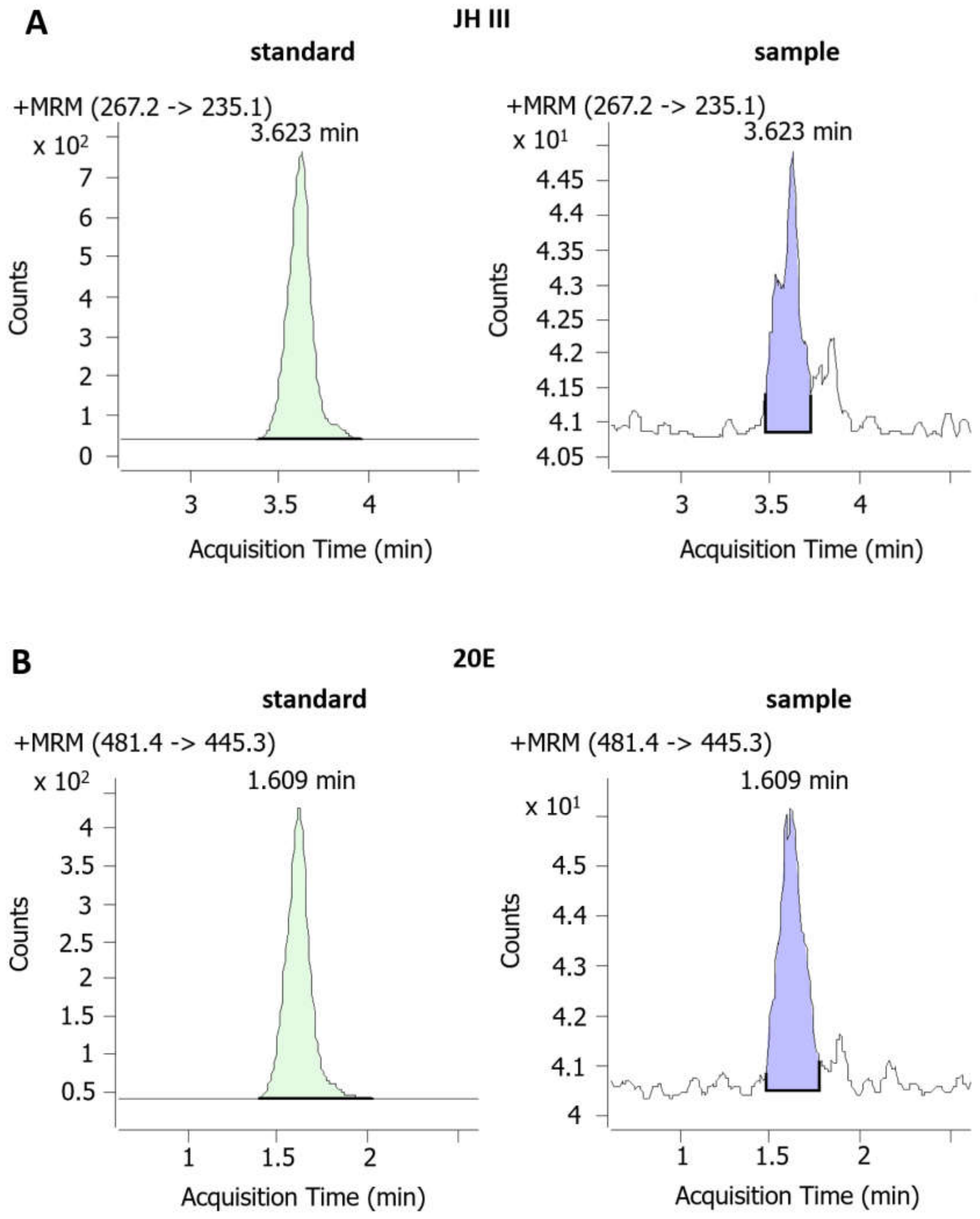

Supplementary Figure 7. The selected reaction monitoring chromatograms of JH III and 20E, respectively.

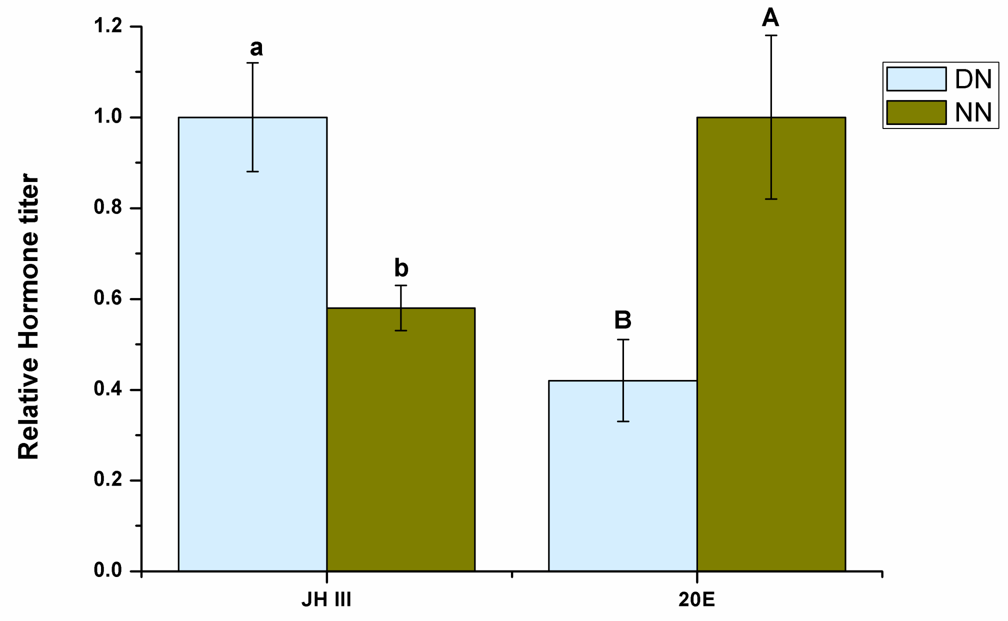

Supplementary Figure 8. The relative hormones titer in diapause and non-diapause nymphs.

Supplementary Table 1 Transcriptome sequencing data assembly statistics tables

Length Range	Contig	Transcript	Unigene
**200-300**	5,220,508	27,455	22,183
**300-500**	17,214	21,393	15,051
**500-1000**	12,884	20,998	11,398
**1000-2000**	9,046	21,275	8,531
**2000+**	6,682,	19,557	6,588
**Total Number**	5,266,334	110,678	63,751
**Total Length**	276,710,777	125,632,068	52,938,652
**N50 Length**	49	2,081	1,585
**Mean Length**	52.54	1135.11	830.40

Supplementary Table 2 Unigenes annotation database

Annotated databases	Unigene	≥300nt	≥1000nt
**NR**	26,934	22,293	11,631
**GO**	10,756	8,574	4,610
**COG**	10,177	8,465	4,779
**KOG**	19,719	16,277	8,936
**KEGG**	9,816	8,179	4,532
**All**	29,217	23,586	11,790

Supplementary Table 3. Proteins identified by TMT and RP HPLC-MS/MS

No.	UniProtKB	Protein name.	Ratio	P value	Interpro ID	DN / NN Regulated
**a1**	R4G8W6	Putative transferrin *[Rhodnius prolixus]*	5.95	0.0300181	IPR001156;IPR018195;	Up
**a2**	A0A0A9YIQ7	Cuticle protein 7 *[Lygus Hesperus]*	2.301	0.0026626	IPR000618;	Up
**a3**	K4K2P2	Cytochrome P450 *[Laodelphax striatella]*	2.141	0.0423416	IPR002403;IPR001128;IPR017972;	Up
**a4**	R4WST1	Starch branching enzyme *[Riptortus pedestris]*	2.0185	0.0236947	IPR006589;IPR014756;IPR006407;IPR013781;IPR013780;IPR013783;IPR006047;IPR006048;IPR004193;IPR017853;	Up
**a5**	R4WCV2	Arginine kinase *[Riptortus pedestris]*	1.862	8.557E-07	IPR022414;IPR022415;IPR022413;IPR014746;	Up
**a6**	A0A023FAR3	4-hydroxyphenylpyruvate dioxygenase *[Triatoma infestans]*	1.851	0.0220695	IPR004360;IPR005956;IPR029068;	Up
**a7**	T1H8C9	Uncharacterized protein *[Rhodnius prolixus]*	1.784	0.0392253	IPR009081;IPR011032;IPR013149;IPR020843;IPR020841;IPR020801;IPR018201;IPR016038;IPR016039;	Up
**a8**	T1I8T4	Uncharacterized protein *[Rhodnius prolixus]*	1.708	0.0008992	IPR016036;IPR016035;IPR016040;IPR001227;IPR013968;IPR014043;IPR014031;	Up
**a9**	T1I3P9	Uncharacterized protein *[Rhodnius prolixus]*	1.633	0.0177421	IPR013035;IPR018091;IPR008210;IPR008209;	Up
**a10**	A0A0A9YQ74	Ras-like protein 2 *[Lygus Hesperus]*	1.61	0.0482782	IPR020849;IPR003579;IPR003578;IPR005225;IPR001806;IPR002041;IPR027417;	Up
**a11**	A0A069DVF9	Dihydrolipoyl dehydrogenase *[Panstrongylus megistus]*	1.552	0.0311142	IPR006258;IPR004099;IPR001327;IPR013027;IPR012999;IPR016156;IPR023753;	Up
**a12**	A0A0A9W6X4	NADP-dependent malic enzyme *[Lygus Hesperus]*	1.5295	0.0354313	IPR015884;IPR016040;IPR012301;IPR012302;IPR001891;	Up
**a13**	A0A023FAP1	Putative g-protein alpha subunit *[Triatoma infestans]*	1.5245	0.0343464	IPR011025;IPR001408;IPR027417;IPR001019;	Up
**a14**	A0A0A9XDT1	Muscle LIM protein Mlp84B *[Lygus Hesperus]*	1.463	0.0006607	IPR001781;	Up
**a15**	R4FNF3	Putative tropomyosin 1 isoform a *[Rhodnius prolixus]*	1.4325	0.0013579	IPR000533;	Up
**a16**	A0A0A9XQG0	Annexin-B11 *[Lygus Hesperus]*	1.43	0.0048788	IPR018502;IPR001464;IPR018252;	Up
**a17**	A0A023FAB6	Putative actin *[Triatoma infestans]*	1.429	0.0351982	IPR022782;	Up
**a18**	D9J128	Putative vacuolar ATP synthase *[Nilaparvata lugens]*	1.417	0.0355857	IPR002842;	Up
**a19**	A0A069DQN8	Putative ubiquinone oxidoreductase ndufs8/23 kDa subunit	1.41	0.012787	IPR010226;IPR017896;IPR017900;IPR001450;	Up

		[Panstrongylus megistus]				
**a20**	A0A069DYD7	Putative titin *[Panstrongylus megistus]*	1.4085	0.0184123	IPR010939;IPR007110;IPR003598;IPR003599;IPR013783;IPR013098;	Up
**a21**	A0A069DXG1	Putative rab subfamily protein of small gtpase *[Panstrongylus megistus]*	1.3955	0.0163522	IPR005225;IPR002041;IPR003579;IPR003578;IPR001806;IPR027417;IPR020849;	Up
**a22**	A0A0K1IJN8	Juvenile hormone acid methyltransferase *[Nilaparvata lugens]*	1.394	0.0148595	IPR011025;IPR027417;IPR001019;IPR000654;	Up
**a23**	A0A069DYH2	Protein required for fusion of vesicles in vesicular transport alpha-snap *[Panstrongylus megistus]*	1.391	0.0020505	IPR000744;IPR011990;	Up
**a24**	A0A069DZK7	V-type proton ATPase subunit a *[Panstrongylus megistus]*	1.376	0.0232392	IPR002490;IPR026028;	Up
**a25**	A0A069DRF5	Triosephosphate isomerase *[Panstrongylus megistus]*	1.3725	0.0030326	IPR013785;IPR020861;IPR000652;IPR022896;	Up
**a26**	A0A069DTM9	Putative vacuolar h+-atpase v1 sector subunit c *[Panstrongylus megistus]*	1.366	0.0014818	IPR004907;	Up
**a27**	V5JDH8	Tropomyosin 2 *[Nilaparvata lugens]*	1.3585	0.0002843	IPR000533;	Up
**a28**	A0A023F5Q0	Putative acetyl-coa carboxylase *[Triatoma infestans]*	1.3575	0.0051286	IPR013785;IPR003379;IPR009057;IPR000891;IPR000089;IPR016185;IPR005479;IPR005481;IPR005482;IPR011054;IPR011053;IPR005930;IPR011761;IPR011764;IPR013816;IPR013815;	Up
**a29**	M9TFX8	V-ATPase B subunit *[Peregrinus maidis]*	1.3495	0.0396123	IPR000194;IPR022879;IPR000793;IPR005723;IPR027417;IPR020003;IPR004100;	Up
**a30**	A0A023F687	Putative vacuolar h+-atpase v1 sector subunit b *[Triatoma infestans]*	1.3425	3.523E-06	IPR000793;IPR000194;IPR027417;IPR005723;IPR020003;IPR022879;IPR004100;	Up
**a31**	A0A0A9ZC53	Four and a half LIM domains protein 2 *[Lygus Hesperus]*	1.341	0.002594	IPR001781;	Up
**a32**	A0A023F9T3	Putative nadp-dependent isocitrate dehydrogenase *[Triatoma infestans]*	1.3395	0.0235394	IPR019818;IPR024084;IPR004790;	Up
**a33**	G8CV15	Glyceraldehyde-3-phosphate dehydrogenase *[Laodelphax striatella]*	1.331	0.006109	IPR006424;IPR016040;IPR020829;IPR020828;IPR020831;IPR020830;	Up
**a34**	A0A0A9VZC2	Putative citrate synthase 2 *[Lygus hesperus]*	1.3245	0.0114715	IPR019810;IPR016142;IPR016141;IPR010109;IPR002020;	Up

**a35**	A0A069DWY8	Putative rna-binding translational regulator irp aconitase superfamily *[Panstrongylus megistus]*	1.316	0.0101354	IPR006248;IPR000573;IPR001030;IPR018136;IPR015928;IPR015931;IPR015932;	Up
**a36**	A0A069DXN9	Phosphoinositide phospholipase *[Panstrongylus megistus]*	1.31	0.0375653	IPR015359;IPR000909;IPR000008;IPR001711;IPR011992;IPR011993;IPR017946;IPR001192;IPR009535;	Up
**a37**	E5FQV0	Carboxylesterase *[Laodelphax striatella]*	1.3075	0.0052595	IPR029058;IPR002018;IPR019826;IPR019819;	Up
**a38**	A0A069DPC3	Putative ribosomal protein l27ae *[Panstrongylus megistus]*	1.3065	1.414E-05	IPR001196;IPR021131;	Up
**a39**	V5RDY5	Enolase *[Nilaparvata lugens]*	1.302	0.0159623	IPR020809;IPR020810;IPR020811;IPR000941;IPR029017;IPR029065;	Up
**b1**	R4G4S6	Putative gtp-binding protein drg2 odn superfamily *[Rhodnius prolixus]*	0.423	0.0358844	IPR027417;IPR012676;IPR004095;IPR005225;IPR006073;IPR006074;	Down
**b2**	R4G8V7	Putative ribosomal protein *[Rhodnius prolixus]*	0.502	0.0127818	IPR011332;IPR000552;	Down
**b3**	A0A0A9X5A5	40S ribosomal protein *[Lygus hesperus]*	0.535	0.0092707	IPR000630;	Down
**b4**	A0A023FAS8	Putative transcription factor *[Triatoma infestans]*	0.5415	0.0440813	IPR016641;IPR002715;IPR000449;	Down
**b5**	R4WCQ3	Elongation factor 2 *[Riptortus pedestris]*	0.5605	0.042667	IPR005225;IPR004161;IPR000640;IPR000795;IPR014721;IPR027417;IPR020568;IPR005517;IPR009000;IPR009022;	Down
**b6**	A0A0A9X1N8	ATP-dependent RNA helicase vasa *[Lygus hesperus]*	0.575	0.0044937	IPR027417;IPR011545;IPR000629;IPR001876;IPR001650;IPR014014;IPR014001;	Down
**b7**	A0A0A9WXZ9	60S ribosomal protein *[Lygus hesperus]*	0.585	1.056E-05	IPR020785;IPR020784;IPR020783;IPR000911;	Down
**b8**	R4WIW8	Elongation factor 1 *[Riptortus pedestris]*	0.608	0.0343169	IPR001326;IPR018940;IPR014717;IPR010987;IPR014038;	Down
**b9**	R4WDS4	Translationally controlled tumor protein *[Riptortus pedestris]*	0.6205	0.0192856	IPR011057;IPR018103;IPR018105;IPR011323;	Down
**b10**	A0A069DQE7	Putative 60s ribosomal protein *[Panstrongylus megistus]*	0.6275	3.767E-06	IPR022803;IPR002132;IPR020929;	Down
**b11**	A0A023F9G7	Histone H2A *[Triatoma infestans]*	0.6335	0.0011983	IPR007125;IPR009072;IPR002119;	Down
**b12**	A0A0A9YSQ6	Cullin-associated NEDD8-dissociated protein *[Lygus Hesperus]*	0.661	0.0022856	IPR016024;IPR011989;IPR013932;	Down
**b13**	R4WHW2	Calreticulin *[Riptortus pedestris]*	0.671	0.0008187	IPR009033;IPR001580;IPR013320;IPR009169;IPR018124;	Down
**b14**	R4FQH4	Putative endoplasmic reticulum glucose-regulated protein	0.672	0.0001886	IPR020575;IPR020568;IPR003594;IPR001404;IPR019805;	Down

		[Rhodnius prolixus]				
**b15**	G8CV16	60S ribosomal protein *[Laodelphax striatella]*	0.68	0.0001136	IPR004038;IPR004037;IPR001921;IPR029064;IPR018492;	Down
**b16**	A0A0A9Z1J5	Uncharacterized protein *[Lygus hesperus]*	0.682	4.471E-05	IPR011057;IPR018103;IPR018105;IPR011323;	Down
**b17**	A0A023FAT4	Putative translationally controlled tumor protein *[Triatoma infestans]*	0.6865	0.0026082	IPR011323;IPR018103;IPR018105;IPR011057;	Down
**b18**	A0A096W1J4	Odorant binding protein 7 *[Laodelphax striatella]*	0.6915	0.0063997	IPR006170;IPR023316;	Down
**b19**	T1HKS4	Uncharacterized protein *[Rhodnius prolixus]*	0.6945	0.0135431	IPR019355;	Down
**b20**	A0A023F571	Putative tata-binding protein-interacting protein *[Triatoma infestans]*	0.697	0.0333032	IPR016024;IPR011989;IPR013932;	Down
**b21**	A0A0A9XK26	40S ribosomal protein *[Lygus hesperus]*	0.697	0.0055179	IPR023591;IPR018130;IPR027498;IPR005707;IPR001865;	Down
**b22**	A0A069DRR7	Putative rna-binding protein musashi/mrna cleavage and polyadenylation factor i complex *[Panstrongylus megistus]*	0.7025	0.0025488	IPR000504;IPR012677;	Down
**b23**	A0A069DZI1	Putative rna helicase *[Panstrongylus megistus]*	0.707	0.0109958	IPR001650;IPR000629;IPR027417;IPR011545;IPR014014;IPR014001;	Down
**b24**	A0A0E3DQZ8	ATP synthase subunit alpha *[Laodelphax striatella]*	0.707	0.0017892	IPR027417;IPR011709;IPR014001;IPR007502;IPR011545;IPR001650;	Down
**b25**	A0A0A9X8I3	26S proteasome non-ATPase regulatory subunit *[Lygus hesperus]*	0.7115	0.0055165	IPR013143;IPR011990;IPR011991;IPR000717;	Down
**b26**	R4WDH0	T-complex protein 1 subunit delta *[Riptortus pedestris]*	0.716	0.0414959	IPR002194;IPR017998;IPR002423;IPR012717;IPR027410;IPR027413;IPR027409;	Down
**b27**	A0A0A9Y2K6	Uncharacterized protein *[Lygus hesperus]*	0.7165	0.0011228		Down
**b28**	E2J7E3	40S ribosomal protein *[Triatoma matogrossensis]*	0.721	5.035E-05	IPR027500;IPR018281;IPR001593;	Down
**b29**	A0A023FB83	Putative 60s ribosomal protein *[Triatoma infestans]*	0.722	0.0083234	IPR001063;IPR018260;IPR005721;	Down
**b30**	A0A023F3Q2	Putative cytochrome *[Triatoma infestans]*	0.7285	0.0409777	IPR001128;IPR002401;IPR017972;	Down
**b31**	M9SV74	Cytochrome P450 CYP314A1 *[Laodelphax striatella]*	0.7385	0.023908	IPR018273;IPR001210;	Down
**b32**	A0A023F8Z5	Putative medium subunit of clathrin adaptor complex *[Triatoma infestans]*	0.7435	0.0245954	IPR011012;IPR028565;IPR022775;IPR008968;	Down

**b33**	M9TNB1	Ribosomal protein *[Peregrinus maidis]*	0.744	0.0007577	IPR018255;IPR016180;IPR001197;	Down
**b34**	A0A0A9W869	Elongation factor 2 *[Lygus hesperus]*	0.746	0.0002765	IPR000795;IPR027417;IPR000640;IPR005225;IPR009000;IPR009022;IPR014721;IPR005517;IPR020568;IPR004161;	Down
**b35**	A0A069DP44	Peptidyl-prolyl cis-trans isomerase *[Panstrongylus megistus]*	0.7525	0.0020296	IPR020892;IPR002130;IPR024936;IPR029000;	Down
**b36**	A0A069DVR6	Putative polypeptide release factor 3 *[Panstrongylus megistus]*	0.753	0.0145326	IPR009001;IPR009000;IPR009818;IPR000795;IPR027417;IPR004160;IPR004161;	Down
**b37**	A0A023FD44	Putative 60s ribosomal protein *[Triatoma infestans]*	0.754	0.0098864	IPR023573;IPR021138;IPR028877;	Down
**b38**	A0A0A9W0T7	60S ribosomal protein *[Lygus hesperus]*	0.754	0.0098864	IPR023573;IPR021138;IPR028877;	Down
**b39**	V5TGF4	Heat shock cognate protein 70 *[Laodelphax striatella]*	0.756	7.711E-07	IPR013126;IPR029048;IPR029047;IPR018181;	Down
**b40**	A0A069DPJ5	Putative gtp-binding adp-ribosylation factor-like protein *[Panstrongylus megistus]*	0.756	0.0057496	IPR005225;IPR024156;IPR027417;IPR006689;IPR006687;IPR003579;	Down
**b41**	E1U339	Prolyl-endylpeptidase *[Eurygaster integriceps]*	0.76	0.0083235	IPR023302;IPR001375;IPR002470;IPR002471;IPR029058;	Down
**b42**	A0A0A9XY83	Eukaryotic translation initiation factor *[Lygus hesperus]*	0.769	0.0006488	IPR027516;IPR008905;IPR011991;IPR000717;	Down
**b43**	A0A023FCX8	Putative 26s proteasome regulatory complex *[Triatoma infestans]*	0.769	0.0002162	IPR003959;IPR003960;IPR005937;IPR027417;IPR003593;	Down

Supplementary Table 4. The information of overlapping up- and down-regulated candidate proteins.

No.	UniProtKB.	Gene ID	Protein name	DN / NN Regulated
**a1**	R4WCV2	c12741	Arginine kinase *[Riptortus pedestris]*	Up
**a2**	A0A0A9XQG0	c9103	Annexin-B11 *[Lygus Hesperus]*	Up
**a3**	A0A0K1IJN8	c7273	Juvenile hormone acid methyltransferase *[Nilaparvata lugens]*	Up
**a4**	V5JDH8	c9785	Tropomyosin 2 *[Nilaparvata lugens]*	Up
**b1**	R4WHW2	c35020	Calreticulin *[Riptortus pedestris]*	Down
**b2**	G8CV16	c30052	60S ribosomal protein *[Laodelphax striatella]*	Down
**b3**	A0A0E3DQZ8	c31170	ATP synthase subunit alpha *[Laodelphax striatella]*	Down
**b4**	M9SV74	c30182	Cytochrome P450 CYP314A1 *[Laodelphax striatella]*	Down
**b5**	A0A0A9W869	c25616	Elongation factor 2 *[Lygus hesperus]*	Down
**b6**	V5TGF4	c36094	Heat shock cognate protein 70 *[Laodelphax striatella]*	Down

Supplementary Table 5 Primers used in this study

Gene ID	Primer sequence(5'- 3')		Amplicon size (bp)
	Forward	Reverse	
	**For RT-PCR and real-time PCR**		
c39290	TCTCCTTCTGGTCCTGATAT	CCTCTACTTCTGGCTCTTCTA	131
c39432	CATCTCCCAGCAGTGTTT	CTGGAGCCTGGAATAGAG	189
c39646	ACTACAACATGAATTGGCAACA	AACGCTCTGAAGGCTGGT	191
c40211	AGAGGTGCTTGCCTTCAT	CAACCCTATCATCGTCTCC	200
c40324	CATCCTGCCCAAACACCT	ATCCGCATCATTGCCCAC	166
c47475	GCTGAGGGTAGCACCGTCTC	GCCAGCAACTCGGGAGACA	194
c43693	GCCCTCAAAGATGAACGG	ACGCCTTGGTGTAGCACA	132
c41099	GGCAACTGAGATGGGATG	TGGGAGCAAGAAGAAACC	116
c39870	ACAATCCTCCAGTTCAGCC	TCTCCCTCCGTTCTCCTT	138
c39251	CCTCAGCATCCCTCACTT	CTTAGCGCCATCTTCCTT	163
c39910	GGGAAGTTTACGGTGGTT	CTCCTCAGAGTCTGCGAGA	168
c41340	ATGTACCGCACTGAGATTC	TGGGCTTACTCCATTGATA	120
c39772	CTGTTCGCAGTCATAGCC	AAGAATCGGGTCAAGGTG	179
c16002	TCACGAAACTACGAGGCT	GGGCGAGAATGTCATAAA	173
c39022	TCCCTACCAGTTCTGTCGT	GCTCTGTTCTCAACCCACC	163
c10069	TTTCAAACCCGCAACATC	AGTACAACACGCCGCAAA	200
c36395	CTACAGCCGCCTCTAATA	GTTCCCTCCCAAACTAAC	194
c31564	TCTCCTGGATGCTTGTTC	AGAAAGTCAGAATGGTGG	116
c30434	ACAATCCTCCATTCTGCA	TTTGAGTCCCAACTACGC	129
c32501	TTCAAGTTGGCAGGAGCAT	AGCCCACCCGAAATCAGT	119
c10990	GGTGCCAGTCGTTTACAG	ATCCAGGACCAACCCATC	125

c26848	AGGGTTGCCTGGTTCATC	CAGCCGCTTTCGCTTCTC	164
c34517	CGTAACAAATCCTCCAAT	GAGCGAGTAATATCAGCAAC	138
c22115	TCAATGTCTTGCCCTCAT	CATCACTCGCATTTCTAAT	184
c23274	TCTGGCAGTGATCGGACAA	TGGCAAATCGGAAACGAC	133
c35294	TACGTTTGGCCCTTTGGT	GCGATTCCTGGTTCCCTC	159
c29504	GGAACTAGGCTATGACCG	CTAGATCGCCGAGTAAGT	158
c33207	ACATCCCAACGACCTTTC	GATGAAGACGGTTGAGGC	117
c35520	ATGGGCACTCCGCTGATG	GCCCTCCTGTTATCGTTACCTT	178
c34143	TGGACGAATCTGACAACA	GTTACAACCCAAACGAAG	160
qJHAMT	TGGACAGATTCATTACACCCTA	TTCTTTGGCATACGACCC	190
qShd	CGGAAAGATGGCTGAGCAACAAC	GCGTTTGGCGGGACACATTC	92
EF-1	CCTTACCCATGTTGGATGCTTATT	TGCTTCTGTCTTCCTCTTTCTTCC	95
ARF	TTGGACAGTATCAAGACCCATC	GCAGCAATGTCATCAATAAGC	104
**For dsRNA synthesis**			
dsJHAMT	ggatcctaatacgactcactataggGTAGTCCATAGTCCGCTCTT	ggatcctaatacgactcactataggGCTTCCCTTCACTTCCTT	452
dsShd	ggatcctaatacgactcactataggCGACCGATACACCAATG	ggatcctaatacgactcactataggTCTCGCTCCTCTGACAAT	470
dsGFP	ggatcctaatacgactcactataggAAGTTCAGCGTGTCCG	ggatcctaatacgactcactataggCTTGCCGTAGTTCCAC	414
